# Behavioral and transcriptomic profiling of mice null for *Lphn3*, a gene implicated in ADHD and addiction

**DOI:** 10.1002/mgg3.207

**Published:** 2016-03-04

**Authors:** Caitlin A. Orsini, Barry Setlow, Michael DeJesus, Stacy Galaviz, Kimberly Loesch, Thomas Ioerger, Deeann Wallis

**Affiliations:** ^1^ Department of PsychiatryMcKnight Brain InstituteUniversity of Florida College of MedicineGainesvilleFlorida32610; ^2^ Department of Computer Science and EngineeringTexas A&M UniversityCollege StationTexas77843; ^3^ Department of Biochemistry and BiophysicsTexas A&M UniversityCollege StationTexas77843

**Keywords:** ADHD, behavior, calcium, cell adhesion, Lphn3/Adgrl3, neurite outgrowth, SUD, transcriptome

## Abstract

**Background:**

The *Latrophilin 3* (*LPHN3*) gene (recently renamed *Adhesion G protein‐coupled receptor L3 (ADGRL3*)) has been linked to susceptibility to attention deficit/hyperactivity disorder (ADHD) and vulnerability to addiction. However, its role and function are not well understood as there are no known functional variants.

**Methods:**

To characterize the function of this little known gene, we phenotyped *Lphn3* null mice. We assessed motivation for food reward and working memory via instrumental responding tasks, motor coordination via rotarod, and depressive‐like behavior via forced swim. We also measured neurite outgrowth of primary hippocampal and cortical neuron cultures. Standard blood chemistries and blood counts were performed. Finally, we also evaluated the transcriptome in several brain regions.

**Results:**

Behaviorally, loss of *Lphn3* increases both reward motivation and activity levels. *Lphn3* null mice display significantly greater instrumental responding for food than wild‐type mice, particularly under high response ratios, and swim incessantly during a forced swim assay. However, loss of *Lphn3* does not interfere with working memory or motor coordination. Primary hippocampal and cortical neuron cultures demonstrate that null neurons display comparatively enhanced neurite outgrowth after 2 and 3 days in vitro. Standard blood chemistry panels reveal that nulls have low serum calcium levels. Finally, analysis of the transcriptome from prefrontal cortical, striatal, and hippocampal tissue at different developmental time points shows that loss of *Lphn3* results in genotype‐dependent differential gene expression (DGE), particularly for cell adhesion molecules and calcium signaling proteins. Much of the DGE is attenuated with age, and is consistent with the idea that ADHD is associated with delayed cortical maturation.

**Conclusions:**

Transcriptome changes likely affect neuron structure and function, leading to behavioral anomalies consistent with both ADHD and addiction phenotypes. The data should further motivate analyses of *Lphn3* function in the developmental timing of altered gene expression and calcium signaling, and their effects on neuronal structure/function during development.

## Introduction

It is well established that attention deficit/hyperactivity disorder (ADHD) and substance use disorders (SUDs) have strong genetic components (Li and Burmeister 2009; Treutlein and Rietschel 2011; Arcos‐Burgos et al. 2012a; Gorwood et al. 2012; Hart et al. 2012), but there is still a paucity of information regarding reliable biomarkers for these diseases, which limits the ability to develop targeted therapies for their treatment. Recently, however, several genetic linkage and candidate gene association studies have identified the *LPHN3* gene (OMIM 616417) as a candidate marker for ADHD and SUDs. Genome‐wide linkage analyses in large multigenerational families have repeatedly shown that variants within the *LPHN3* gene on Chromosome 4q confer risk of susceptibility to the development of ADHD and disruptive behaviors, such as SUDs (Arcos‐Burgos and Muenke 2010; Arcos‐Burgos et al. 2010; Martinez et al. 2011; Ribases et al. 2011). Further analyses show that *LPHN3* variants can be used to better predict ADHD severity (Acosta et al. 2011; Jain et al. 2012), long‐term outcomes (Acosta et al. 2011; Jain et al. 2012), patterns of brain metabolism using proton magnetic resonance spectroscopy (1H‐MRS) (Arcos‐Burgos et al. 2010, 2012b; Jain et al. 2012), and response to treatment (Acosta et al. 2011; Jain et al. 2012). As far as we are aware, these LPHN3 linkages and associations represent the most robustly replicated genetic findings for ADHD (Arcos‐Burgos et al. 2010; Acosta et al. 2011; Ribases et al. 2011; Choudhry et al. 2012; Jain et al. 2012; Labbe et al. 2012). Vulnerability to addiction is a complex trait with strong genetic influences that are largely shared by abusers of multiple legal and illegal addictive substances (Arcos‐Burgos et al. 2012a). Both linkage and association‐based genome scans have been conducted for specific drug dependencies as well as for vulnerability to addiction. While independently each study has identified many different loci, multiple independent studies converge at *LPHN3*. Initially, alcohol abuse was linked to Chromosome 4 in the area of *LPHN3* utilizing two‐point linkage analysis in families with alcohol dependence to identify a nominally significant locus at D4S244 (Reich et al. 1998), which is just under 2.5‐Mb downstream of *LPHN3*. Additional evidence is supplied by another study that identified three *LPHN3* SNPs associated with SUD (Liu et al. 2006). These *LPHN3* SNPs, as well as additional *LPHN3* SNPs, have been replicated in several different populations, including alcohol‐dependent samples from the United States (Bergen et al. 1999; Johnson et al. 2006; Liu et al. 2006) and methamphetamine‐dependent samples from Japan and Taiwan (Uhl et al. 2008a,b).

*LPHN3* is a member of the latrophilin subfamily of G‐protein‐coupled receptors (GPCRs) (Martinez et al. 2011). LPHN1 and LPHN2 were initially discovered as receptors for *α*‐latrotoxin (a major component of black widow spider venom), which, when bound to LPHNs, causes exocytosis of neurotransmitters (Krasnoperov et al. 1997; Sugita et al. 1998; Rahman et al. 1999; Bittner 2000; Sudhof 2001; Volynski et al. 2004). Structurally, LPHNs appear to be chimeras between cell surface receptors and intracellular signaling molecules (Krasnoperov et al. 2002). Latrophilins have seven transmembrane regions as well as large extracellular and intracellular domains. The long N‐terminal extracellular sequence contains a SUEL LECTIN domain, an olfactomedin‐like domain, a homology region with BAI 1‐3, and a cysteine‐rich GPCR proteolysis site (Martinez et al. 2011). Until recently, the endogenous ligands for LPHN receptors were unknown and any information about the function of these receptors was derived from studies using *α*‐latrotoxin as the ligand for LPHN1 and 2. The antiparasitic drug emodepside has also provided insight into the function of latrophilins in *C. elegans* (Willson et al. 2004; Harder et al. 2005). In the last several years, however, fibronectin leucine‐rich transmembrane protein 3 (FLRT3) has been identified as an endogenous postsynaptic ligand for LPHN3 (O'Sullivan et al. 2012). FLRT family members may function in cell adhesion and/or as signaling receptors, as structurally they resemble small leucine‐rich proteoglycans found in the extracellular matrix. Interference with this interaction reduces excitatory synapse density in cultured neurons, and decreases afferent input strength and dendritic spine number in dentate granule cells, indicating that LPHN3 and its ligand FLRT3 play an important role in glutamatergic synapse development (O'Sullivan et al. 2012).

LPHNs may activate several different signaling pathways (for review see Lesch et al. (Lesch et al. 2013)). As GPCRs, they can interact with G*α*q to stimulate both phospholipase C and inositol‐3‐phosphate, resulting in calcium mobilization from intracellular Ca^2+^ stores (Davletov et al. 1998; Ichtchenko et al. 1999). In addition, the long C‐terminal domain of LPHN3 interacts with SHANK proteins (Kreienkamp et al. 2000), which interact with a variety of additional proteins important for neuron function and growth. Indeed, SHANK3 mutations trigger modification of dendritic spine morphology (Durand et al. 2012) and defects in striatal synapses and cortico‐striatal circuits (Peca et al. 2011). Hence, loss of LPHN3 function may result in altered GPCR and calcium signaling, and possibly even impact neuron structure and function.

Another group has evaluated the role of the *lphn3.1* ortholog in zebrafish development, and demonstrated that loss of *lphn3.1* causes a reduction and misplacement of dopamine‐positive neurons in the ventral diencephalon and a hyperactive/impulsive motor phenotype that is rescued by the ADHD treatment drugs methylphenidate and atomoxetine (Lange et al. 2012). In addition, using a *Lphn3* null mouse model, previous work from our labs showed that *Lphn3* null mice have significantly higher dopamine and serotonin levels in the dorsal striatum than their wild‐type (WT) littermates and are significantly more active than WT mice in an open field (Wallis et al. 2012). In addition, comparison of gene expression revealed differential expression of dopamine and serotonin receptors and transporters, neurotransmitter metabolism genes, and neural developmental genes between *Lphn3* null and WT littermates (Wallis et al. 2012). This study extends this phenotyping of *Lphn3* null mice with behavioral assessments of reward motivation, working memory, and motor function. In addition, biochemical, structural, and transcriptomic assays were used to further characterize the effects of loss of *Lphn3* function. Together, these findings reveal a more in depth characterization of *Lphn3* function and provide clues as to how polymorphisms in this gene could mediate psychiatric disorders such ADHD and SUDs.

## Materials and Methods

### Ethical compliance

Animal studies were carried out in accordance with the Texas A&M University Institutional Animal Care and Use Committee (IACUC 2012‐062 and 2015‐0025) and the University of Florida Institutional Animal Care and Use Committee (IACUC #201207354). All animal procedures were carried out in accordance with the National Institutes of Health Guide for the Care and Use of Laboratory Animals.

### Subjects

Mice originated from a colony of *Lphn3* (129S4/Sylvae and C57 mixed background) (Gene ID: 319387) mutant mice maintained by heterozygous matings. All animals were genotyped from genomic DNA isolated from tails collected at weaning using Extract‐N‐Amp (Sigma Aldrich, St. Louis, MO). Details regarding the genotype, age, and sex of mice in all cohorts are provided in Table S1. Mice that were tested in the food motivation and working memory tasks were singly housed in order to closely monitor food intake and body weight, and maintained on a 12 h light/dark cycle (on at 7:00) with free access to water at all times. During behavioral testing, these mice were food‐restricted to 85–90% of their free‐feeding body weight with their target weights adjusted upward every week by 1 g to account for growth. For the remaining behavioral assays and molecular experiments, mice were housed in groups of 5/cage in an animal room at 20–22°C, under a 12‐h light/dark cycle (on at 7:00) with ad libitum access to food and water. Mice were killed by CO_2_ asphyxiation and cervical dislocation prior to tissue collection.

### Instrumental responding for food reward on ascending fixed ratio schedules

Testing for food motivation took place in four identical computer‐controlled operant test chambers (Coulbourn Instruments, Harvard Biosciences, Holliston, MA). The chambers were housed in sound‐attenuating cabinets and were outfitted with a recessed food pellet delivery trough with a photobeam to detect nosepokes and a 1.12 W lamp to illuminate the food trough. The trough was located 5 cm above the floor in the center of the front wall of the chamber. A pellet feeder was positioned to deliver 20 mg food pellets (formula 5TUL, Test Diet) into the trough. A retractable lever was located either to the left or right of the trough (counterbalanced across genotypes), 9.5 cm above the floor of the chamber. A 1.12 W houselight was mounted on the rear wall of the sound‐attenuating cabinet, and remained on for the duration of testing. The test chambers were interfaced with a computer running Graphic State 3.0 software (Coulbourn Instruments, Harvard Biosciences), which controlled task event delivery and data collection. Behavioral procedures were identical to those used previously in our lab to assess motivation to work for food reward (Mendez et al. 2009). Mice (4–6 month, *n* = 15 null, six male/10 female and *n* = 18 wild type, eight male/10 female) initially underwent one session of magazine training, followed by five daily sessions in which a single lever press resulted in delivery of a single food pellet into the food trough (i.e., responses on the lever were reinforced on a fixed‐ratio 1 (FR1) schedule). In subsequent test sessions, the response ratio required to earn a food pellet was increased to FR3, FR10, FR20, and FR40 (one session/day).

### Delayed response working memory task

Working memory was assessed in the same operant test chambers used to test food motivation, except that two retractable levers (located to both the left and right of the food trough) were used in each chamber. A subset of the mice used for the food motivation task (4–6 month, *n* = 4 null, two male/ two female and *n* = 7 wild type, three male/ four female) was tested immediately afterward in the delayed response task. The design of the task was based on (Sloan et al. 2006) and (Beas et al. 2013). Each session was 40 min in duration, and the house light was illuminated throughout the entire session except during timeout periods (see below). Each trial began with insertion of a single lever (the “sample” lever) into the chamber. The left/right position of this lever was pseudorandomly selected within each pair of trials, and a lever press caused it to retract and started the delay period timer. During the delay, mice were required to nosepoke into the food trough, and the first nosepoke after the delay timer expired initiated the “choice” phase of the trial. During the choice phase, both levers were extended, and a response on the same lever pressed during the sample phase (a correct response) resulted in both levers being retracted and delivery of a single 20 mg food pellet. Entry into the food trough to collect the food pellet initiated a 5 sec intertrial interval, after which the next trial was initiated. A response on the opposite lever from that chosen during the sample phase (an incorrect response) resulted in both levers being retracted and initiation of a 5 sec “timeout” period during which the house light was extinguished, followed immediately by the start of the next trial.

Once mice were shaped on the various components of the task (e.g., lever shaping, nosepoking), a set of seven delays was introduced. The presentation of delay durations was randomized within each block of seven trials. In the first three sets of delays, some of the delays were repeated within the session to ensure that delays were not introduced too rapidly. Upon establishing >75% correct performance across two consecutive sessions at a given set of delays, mice were advanced to the next set (Set 1: 0, 0, 1, 1, 2, 2, 3 sec; Set 2: 0, 1, 2, 2, 3, 3, 4 sec; Set 3: 0, 1, 2, 3, 4, 4, 5 sec; Set 4: 0, 1, 2, 3, 4, 5, 6 sec; Set 5: 0, 2, 4, 6, 8, 10, and 12 sec). Mice were tested for five consecutive sessions on the delays in Set 5.

### Rotarod test

A separate cohort of mice was tested on a rotarod (Ugo‐Basile, Model No. 7750, Comerio, Italy) to evaluate motor coordination. On three separate trials (15 min intertrial interval), mice (3–6 month, *n* = 9 null, four male/ five female and *n* = 8 wild type, three male/ five female) were placed on the rotarod apparatus with a starting speed of 4 rpm. The rotation speed was increased from 4–40 rpm over 5 min, and latency to fall was measured.

### Forced swim test

A separate cohort of mice was assessed in a forced swim test to evaluate depressive‐like behavior. Using a protocol adapted from (Castagne et al. 2010), mice (3–6 month, *n* = 11 null, four male/ seven female and *n* = 10 wild type, three male/ seven female) were placed in a 4 L glass beaker filled with 3 L of water (23–25°C) for 6 min. Latency to immobility (defined as a period of at least 1 sec without any active escape behavior) was evaluated during the first 2 min of the test because the latency to the first immobility improves the predictive validity of the task (Castagne et al. 2009). Immobility time was evaluated during the final 4 min because animals typically show more stable levels of immobility during this time period (Castagne et al. 2010).

### Primary neuron culture

Timed heterozygous matings were set and E16.5 brains were genotyped and dissected into hippocampus and cortex in ice‐cold Modified Hank's Balanced Salt solution supplemented with 30% glucose, glutamine, sodium pyruvate, and HEPES as described by Fath et al. (2009). Tissue from the entire cortex or hippocampus was pooled based on genotype for each litter. Neurons were dissociated by dicing and incubating with trypsin for 5 min at 37°C, triturating, and were then resuspended in 10% FBS DMEM with glutamine and antibiotics, and strained. Neurons were pelleted and resuspended in B27/DMEM with glutamine and antibiotics. Neurons were counted and plated into 96‐well plates previously coated with 50 *μ*g/mL PDL at approximately 5 × 10^4^ per 96‐well plate.

### Neurite outgrowth

To assess the neurite outgrowth, 96‐well neuron cultures were fixed in 4% PFA at 24, 48, or 72 h and stained with FITC‐conjugated Class III *β*‐tubulin and DAPI. Neurons were imaged on a GE INCell Analyzer using a 20X objective. Four randomly selected fields per well were captured. The GE INCell Developer software was used to analyze the images for overall FITC intensity. FITC intensity was normalized by the DAPI cell count for each field. At least 28 fields of view per genotype were analyzed at each time point (1, 2, and 3 days in vitro).

### Blood collection for chemistry, complete blood count, and manual differential

Nonfasted mice (*n* = 5 null female, 4–5 month) were killed, and blood and serum were immediately collected and processed for chemistry, complete blood count (CBC), and manual differential. Blood chemistries were tested on a Beckman Coulter Olympus AU400e Automated Chemistry‐Immuno Analyzer using Beckman Coulter reagents. CBC was performed on an Abbott Cell‐Dyn 3700 automated hematology analyzer. The white blood cell (WBC) differential, which measures the percentage of each type of WBC, was performed manually on a modified Wright's stained blood smear. Null values were initially compared to 90% Confidence Intervals defined through the analysis of 47 WT mice of the same strain. The WT cohort was comprised of 29 mice from our colony ranging in age from 2.5 to 7 months (five male, 24 female) and an additional eighteen 2–3‐month‐old mice of the same strain of undetermined sex.

### Serum calcium and vitamin D measurements

A second, independent cohort of nonfasted mice was utilized to further evaluate both serum calcium levels and vitamin D concentrations. Serum was collected from mice (4–8‐month‐old; *n* = 10 null, three male/ seven female, *n* = 7 WT, four male/ three female). Serum calcium was tested on a Beckman Coulter Olympus AU400e Automated Chemistry‐Immuno Analyzer using Beckman Coulter reagents. Vitamin D concentrations were determined using Immunodiagnostic Systems 25‐Hydroxy Vitamin D EIA (IDS #AC‐57F1).

### Brain dissections for transcriptome studies

Brains were microdissected from a separate naïve cohort of male null and WT mice at 4 day, 28 day (1 month), and 6 month as described by Chiu et al. (2007). The prefrontal cortex, striatum, and hippocampus were collected and submerged in RNAlater. Tissues from 2–3 mice per time point and region were pooled into each sample, and RNA was extracted using a Qiagen RNEasy miniprep kit (Hilden, Germany). A quantity of 2 *μ*g of total RNA was subjected to poly(A) mRNA capture using magnetic oligo‐dT beads. Captured mRNA was prepared for NGS sequencing using a KAPA Stranded mRNA‐Seq Kit (KAPA Biosystems, Wilmington, MA).

### Primary processing of Illumina RNA‐Seq reads

The RNA‐Seq samples were sequenced on an Illumina 2500 sequencer operated in single‐end mode with a read‐length of 100 bp. Eighteen sequence files were generated in Fastq format using Casava base‐calling software. Each file corresponded to a different combination of genotype, brain tissue, and time point from which the RNA originated. The median number of reads per sample was 31524686. Reads were then mapped to the *Mus musculus* genome (build mm 10) using Bowtie 2.0, generating output files in SAM format. The abundance of reads mapping to each exon of each transcript was tabulated based on their coordinates in the .sam files. Finally, RPKM values (reads per kilobase per million fragments) were calculated by dividing the reads per transcript by the total length (of exons) in nucleotides and total reads in the sample, and then multiplying by 10^9^. For genes with multiple transcripts, the transcript with the maximal RPKM was chosen as representative. The mean number of reads per sample mapping to exons was 14073701, and the median RPKM was 2.0.

### Statistical analyses

Instrumental responding was analyzed using a repeated measures ANOVA, with genotype and sex as between‐subjects variables and FR schedule as a within‐subjects variable. In the delayed response working memory task, data averaged across all Set 5 sessions were analyzed using a repeated measures ANOVA, with genotype as a between‐subjects variable and delay duration as a within‐subjects variable. The number of sessions required to reach criterion performance at each set of delays and the number of trials completed in Set 5 were compared using unpaired *t*‐tests. Sex was not analyzed as a factor due to the small number of males and females within each genotype. For the rotarod test, latencies to fall were analyzed using a repeated measures ANOVA, with genotype and sex as between‐subjects variables, and trial as a within‐subjects variable. In the forced swim test, latency to immobility during the first 2 min of the test and immobility during the last 4 min of the test were analyzed using two‐factor ANOVAs (genotype × sex). Neurite outgrowth was analyzed using two‐factor ANOVA for each brain region, with day and genotype as between‐subjects factors. For blood chemistry, complete blood count, and manual differentials, 90% confidence intervals (CIs) were determined by analysis of 47 strain‐matched wild‐type blood samples from male and female mice aged 2–7 month using Reference Value Advisor V2.1. The CIs were determined using a bootstrap method. These CIs were used to evaluate the first cohort of null mice. As an additional test, the first cohort of null mice was also compared using an unpaired *t*‐test to six age, sex and strain‐matched WT mice. Following this analysis, a second cohort was established and evaluated for both serum calcium levels and vitamin D using unpaired *t*‐tests.

### Analysis of differential gene expression (DGE) and RNA splicing

The samples were analyzed for DGE using DESeq (version 2.0; Bioconductor, Seattle, WA). A model comparing expression levels in WT versus null mice was constructed, treating the samples representing different brain regions and time points as replicates. Models were also built conditioned on genotype and brain region (treating different time points as replicates), and conditioned on genotype and time point (treating different brain regions as replicates). For each comparison performed, the genes were sorted by adjusted *P*‐value (with the Benjamini–Hochberg FDR correction for multiple tests applied), and *P*‐adj < 0.05 was used as a cutoff to identify statistically significant up‐ and downregulated genes.

To evaluate differential RNA processing, we reprocessed the raw data to count reads mapping to splice junctions. The raw ~100 bp reads were split into thirds, and the left and right portions were remapped independently to the genome. Sequence alignment was used to look for gaps representing introns. Finally, we compared all WT samples to all null samples to define which transcripts have genotype‐based differences in alternative splicing. Chi‐square analysis was used to identify splice sites where the distribution of connections differed significantly between WT and null mice, for sites represented by at least 10 reads in both samples.

## Results

### Instrumental responding for food reward on ascending fixed ratio schedules

**Figure 1:**
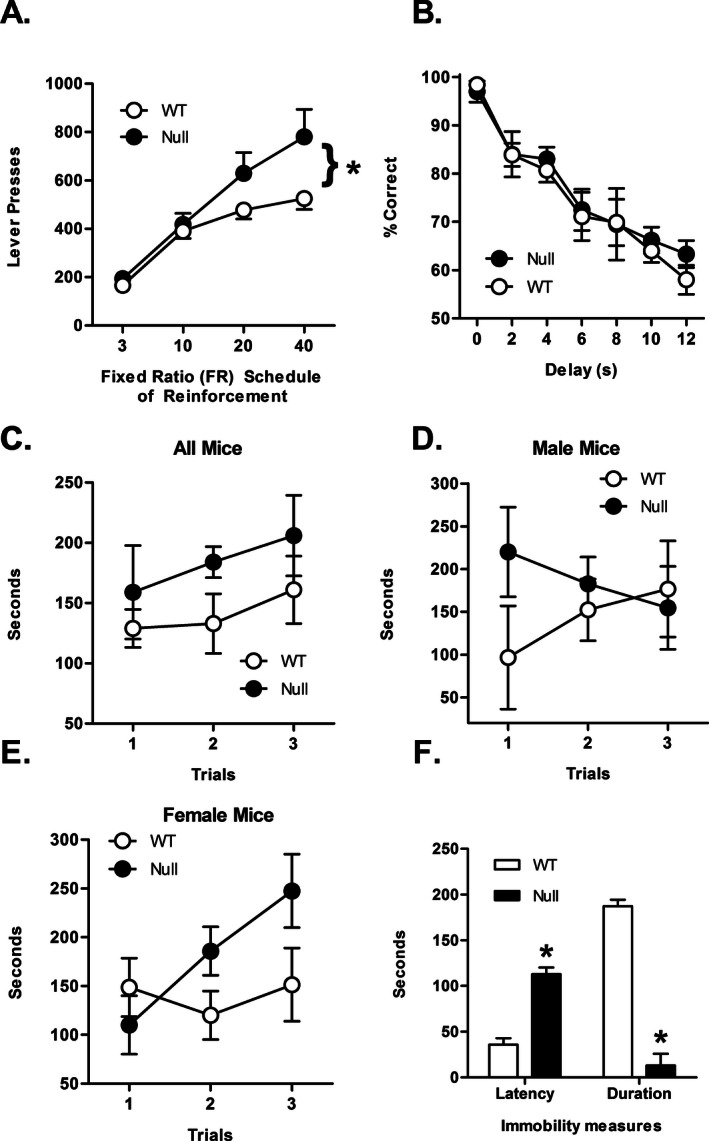
Behavioral characterization of *Lphn3*‐mutant mice and WT controls. (A) All mice were tested under a series of fixed ratio schedules of lever pressing for food reward (FR3, FR10, FR20, and FR40), one schedule/day for 5 days. There was a significant interaction between genotype and schedule, such that null mice responded more than WT mice on the high response ratio schedules. (B) A subset of mice was tested in a delayed response working memory task. While there was a significant main effect of delay as evidenced by the decrease in accuracy as the delay duration increased, there were no differences in accuracy between null and WT mice. (C) A separate cohort of mice was tested on the rotarod to assess motor coordination. There were no differences between null and WT mice in the length of time they remained on the rod. (D) Performance on the rotarod only in male mice. There were no differences between null and WT males in the length of time they remained on the rod. (E) Performance on the rotarod only in female mice. There were no differences between null and WT females in the length of time they remained on the rod. (F) Another cohort of mice was evaluated in the forced swim test in which latency to immobility and duration of immobility were assessed. Null mice took significantly longer to become immobile during the first 2 min of the session and spent significantly less time immobile in the last 4 min of the session compared to WT mice. Asterisks indicate a *P*‐value < 0.05.

A two‐factor, repeated measures ANOVA revealed a significant main effect of FR schedule such that mice pressed more on higher FRs [*F* (3, 96) = 57.70, *P *<* *0.001] and a significant interaction between FR schedule and genotype [*F* (3, 96) = 3.45, *P *=* *0.02] such that *Lphn3* null mice made more lever presses than WT controls at higher FR schedules (Fig. 1A), suggestive of an increase in appetitive motivation for food reward. This effect was independent of sex, as there was neither a schedule × sex interaction [*F* (3, 90) = 0.29, *P *=* *0.84] nor a schedule × genotype × sex interaction [*F* (3, 90) = 0.24, *P *=* *0.87]. In addition, there was no main effect of genotype on the number of entries into the food trough [*F* (1, 31) = 0.08, *P *=* *0.78], although there was a trend toward an interaction between genotype and FR schedule [*F* (3, 93) = 2.70, *P *=* *0.05]. These latter data suggest that the greater lever pressing in null mice was not due to either increased consummatory motivation or a general increase in exploratory activity. Similarly, there were no body weight differences between null and WT mice [*t* (32) = 0.29, *P *=* *0.77], indicating that differences in levels of food restriction likely did not account for the increased lever pressing in null mice.

### Delayed response working memory task

A subset of mice tested in the food motivation task was subsequently tested in the delayed response working memory task. Because of the small numbers of mice, sex differences were not evaluated in this experiment. There was no genotype difference in the number of shaping sessions required to reach the longest set of delays in the task (mean [SEM]: null = 27.5 [3.86], WT = 21.4 [1.72], *t* (9) = 1.67, *P *=* *0.13). Similarly, a two‐factor repeated measures ANOVA performed on choice accuracy data averaged across the five sessions at the longest set of delays (Fig. 1B) revealed a significant main effect of delay (such that accuracy decreased as a function of delay duration [*F* (6, 54) = 34.48, *P *<* *0.001]), but neither a main effect of genotype [*F* (1, 9) = 0.16, *P *=* *0.70] nor a genotype × delay interaction [*F* (6, 54) = 0.27, *P *=* *0.95]. Furthermore, there was neither a main effect of genotype [*F* (1, 9) = 0.18, *P *=* *0.69] nor a significant genotype × session interaction [*F* (4, 36) = 0.92, *P *=* *0.45] on the number of trials completed across the five sessions (mean [SEM] trials completed: null = 121.4 [9.1], WT = 125.4 [5.2]). During the five sessions at the longest set of delays, there were also no differences between null and WT mice in their body weights [*t* (9) = 0.2, *P *=* *0.84].

### Rotarod test

A separate cohort of mice was evaluated in the rotarod assay. Mice were scored for how long they were able to stay on the rod without doing a passive rotation in three separate trials. There was neither a main effect of genotype [*F* (1, 13) = 2.44, *P *=* *0.14] nor a significant trial × genotype interaction [*F* (2, 26) = 0.03, *P *=* *0.98] on time spent on the rod (Fig. 1C). Although there was no main effect of sex [*F* (1, 13) = 0.01, *P *=* *0.91], genotype × sex interaction [*F* (1, 13) < 0.01, *P *=* *0.96] or sex × trial interaction [*F* (2, 26) = 0.93, *P *=* *0.41], there was a significant sex × trial × genotype interaction [*F* (2, 26) = 4.46, *P *=* *0.02], such that null female mice increased their time spent on the rod across successive trials while male null mice decreased their time on the rod across successive trials, an effect that was not observed in WT mice. When analyzed separately by sex (Fig. 1D and E), however, there was neither a main effect of genotype [males, *F* (1, 5) = 0.65, *P *=* *0.46; females, *F* (1, 8) = 2.47, *P *=* *0.16] nor a significant trial × genotype interaction [males, *F* (2, 10) = 2.04, *P *=* *0.18; females, *F* (2, 16) = 2.58, *P *=* *0.11].

### Forced swim test

A separate cohort of mice was assayed in the forced swim test. The first 2 min were evaluated for latency to immobility and the last 4 min were evaluated for time spent immobile. Of the 11 null mice, only one ever became immobile; all other null mice spent the entire test swimming. A multivariate ANOVA revealed that null mice took a significantly longer time to become immobile within the first 2 min [*F* (1, 17) = 66.02, *P < *0.001] and spent significantly less time immobile in the last 4 min [*F* (1, 17) = 117.75, *P *<* *0.001; Fig. 1F]. Importantly, there was no main effect of sex on latency to immobility [*F* (1, 17) = 0.34, *P *=* *0.57] or duration of immobility [*F* (1, 17) = 0.03, *P *=* *0.86]. Similarly, there was no genotype × sex interaction on latency [*F* (1, 17) = 2.74, *P *=* *0.12] or duration measures [*F* (1, 17) = 1.10, *P *=* *0.31].

### Neurite outgrowth

**Figure 2:**
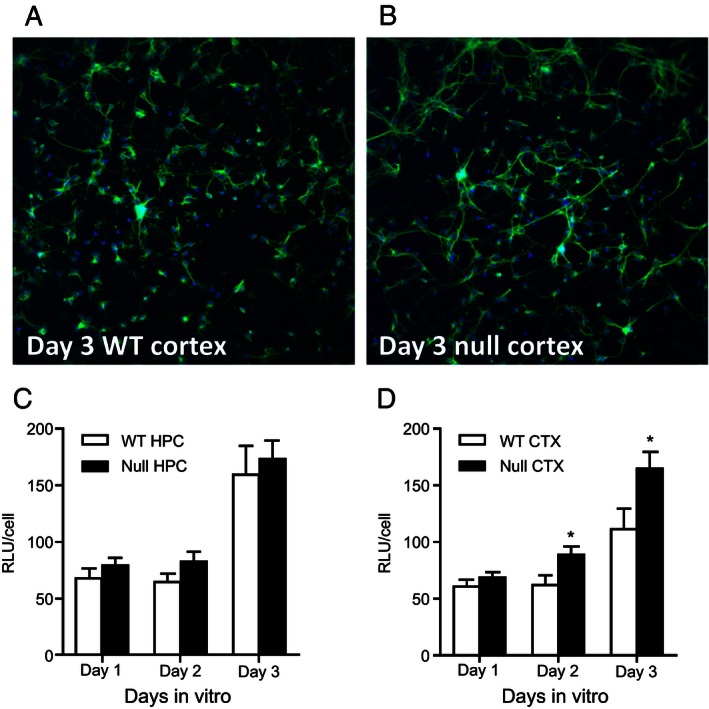
Loss of *Lphn3* leads to enhanced neurite outgrowth. (A) Day 3 in vitro neurites from WT primary cortical cultures. Nuclei are stained blue with DAPI and neurites are labeled with FITC‐conjugated B‐III tubulin. (B) Day 3 in vitro neurites from null primary cortical cultures stained as in A. (C) Quantitation of hippocampal (HPC) neurite staining (FITC) normalized by cell number over days 1–3 in vitro culture from *Lphn3* null and WT mice. (D) Quantitation of cortical (CTX) neurite staining (FITC) normalized by cell number over days 1–3 in vitro culture from *Lphn3* null and WT mice. Asterisks indicate a significant difference between WT and null at the timepoint indicated; *P* < 0.05.

WT and null primary hippocampal and cortical neurons were cultured in 96‐well plates and fixed after 1, 2, or 3 days in vitro. Figure 2 depicts representative Day 3 neurons from WT and null cortex. DAPI counts for each field shown are 284 and 292 cells, respectively, indicating similar cell densities. Overall, FITC intensity normalized by cell count was higher in the null neurons, indicating that there are many more neurites. The figure also shows that the neurites were of increased length and appear more highly branched. Statistical analyses confirmed this impression, revealing greater normalized FITC intensity in null compared to WT cortical neurons (*F* (1, 394) = 12.46, *P *<* *0.001), as well as an increase in intensity across days (*F* (2, 394) = 27.50, *P *<* *0.001) and a trend toward an interaction between genotype and day (*F* (2, 394) = 2.40, *P *=* *0.09; Fig. 2D). In contrast to cortical neurons, in hippocampal neurons, there was neither a main effect of genotype on normalized FITC intensity (*F* (1, 339) = 2.19, *P *=* *0.14) nor a genotype × day interaction (*F* (2, 339) = 0.05, *P *=* *0.95), although there was still an increase in intensity across days (*F* (2, 339) = 34.72, *P *<* *0.001).

### Blood chemistry, complete blood counts, and manual differential

We initially performed standard CBC and blood chemistry panels on samples from null mice (Table S2). Experimental values from null mice were compared to 90% confidence intervals (CI) set by analyses of 47 strain‐matched WT mice. Most notably, average calcium levels for nulls (9.38 mg/dL) fell below the WT CIs (9.50–10.63 mg/dL) due to four of five null mice having low calcium levels. There were some additional values (total bilirubin and aspartate aminotransferase) that fell out of range when all five null mice were averaged, but these were due to variance from only one mouse. As mouse sex and age differences may result in varying calcium levels, as an added control, we compared null values for all measures to values from six age‐ and sex‐matched WT littermates (Table S2). Unpaired t‐tests revealed that only the calcium levels differed between genotypes [*t* (9) = 2.69, *P *<* *0.05]. The average calcium level for these WT littermates was 9.96 mg/dL.

In efforts to validate our finding of low serum calcium levels in null mice, we generated a second, independent cohort of age‐ and sex‐matched mice (*n* = 7 WT and *n* = 10 null) in which we specifically evaluated serum calcium levels. A two‐tailed t‐test confirmed that *Lphn3* null mice have lower serum calcium levels compared to WT mice (mean [SEM]: null = 8.7 [0.23] mg/dL, WT = 9.6 [0.36] mg/dL; *t* (15) = 2.24, *P *=* *0.04). As vitamin D deficiency is the most common cause of low serum calcium, we used an enzyme immunoassay to measure 25‐hydroxy vitamin D3 in serum from this same cohort of mice. We were unable to detect differences between null and WT mice (mean [SEM]: null = 135.5 [3.9] mmol/L, WT = 135.8 [4.4] mmol/L; *t* (15) = 0.07, *P *=* *0.95), indicating that vitamin D deficiency is an unlikely cause of the low serum calcium levels in the *Lphn3* null mice.

### Transcriptomics

WT and null brains were dissected into three brain regions at each of three time points (Table S3). We pooled tissues from 2–3 male mice for each time point and brain region. Specific time points were chosen to reflect different stages of brain development. Postnatal day 4 reflects an early time point when the mouse brain is rapidly developing, and is comparable to a time point prior to when ADHD might be diagnosed in humans. Four weeks of age (P28 or 1 month) was selected as an adolescent time point as it reflects when a human patient would show symptoms of ADHD. Six months of age represents mature adulthood, and though controversial in the literature, might reflect when human ADHD symptoms are ameliorated or lessened. We collected tissue from the hippocampus (important for learning and memory), the prefrontal cortex (essential for executive functions), and the striatum (a primary location of dopaminergic reward‐processing circuitry), all of which are brain regions known to play roles in ADHD. Collectively, this provided 18 samples (each consisting of tissues from 2–3 male mice of the same brain region, age, and genotype) for transcriptome analysis. Data analysis focused on differential changes in transcription between genotypes, both overall and broken down by brain regions and time points.

RPKM values for each transcript were determined by mapping reads to the mouse genome build 10. DeSeq2 was used to calculate DGE levels by evaluating the ratio of WT versus null values for every gene. For each comparison performed, the genes were sorted by adjusted *P*‐value (with the Benjamini–Hochberg FDR method applied). For the overall comparisons of all WT versus all null samples (collapsed across age and brain region), we identified six genes whose overexpression in the nulls was statistically significant and five genes whose underexpression was statistically significant (Table 1). Overexpressed genes include two RIKEN cDNAs with unknown functions, Cox18 (a cytochrome c oxidase assembly protein important for assembly of complex IV of the mitochondrial respiratory chain), Atp10d (which belongs to a subfamily of P‐type ATPases implicated in phospholipid translocation from the exoplasmic to the cytoplasmic leaflet of cellular biological membranes, and which has been associated with atherosclerotic indices (Kengia et al. 2013)), and Ociad1 (a cancer‐specific protein that was identified by immunoscreening of an ovarian carcinoma cDNA expression library (Luo et al. 2001)). Interestingly, protocadherins, which are calcium‐dependent cell–cell adhesion molecules, were both up‐ and down‐regulated (Pcdhgb8 and Pcdhb9, respectively) in *Lphn3* null mice. Underexpressed genes also include Gm 5089 (function unknown), Rpl29 (a cell surface heparin/heparan sulfate‐binding protein (Rohde et al. 1996)), Rplp0 (a ribosomal protein that is a component of the 60S subunit that localizes to the cytoplasm), and Nsun7 (an RNA methyltransferase associated with male sterility). Interestingly, Rpl29 is the only gene that showed altered (decreased) gene expression in all tissues and at all timepoints in the nulls. Rpl29 functions as an important regulator of global growth by modulating the rate of protein synthesis. Homozygous Rpl29 null mice exhibit globally delayed growth around mid‐gestation, including delayed digit and incisor formation, low birth weights associated with a proportional reduction in organ and skeletal size, delayed postnatal development and sexual maturation, and significant postnatal lethality.

**Table 1:** DGE for all WT and all null samples

	Gene	Refseq	Annotation	Fold change	*P*adj
Overexpressed in null	Cox18	NR_028088	Cytochrome c oxidase assembly protein 18	1.61	9.87E‐16
	1500015A07Rik	NR_029432	RIKEN cDNA 1500015A07 gene	1.58	3.91E‐05
	Atp10d	NR_003966	ATPase, class V, type 10D	2.28	1.78E‐04
	C330024D21Rik	NR_015582	RIKEN cDNA C330024D21 gene	4.13	3.95E‐04
	Pcdhgb8	NM_033580	Protocadherin gamma subfamily B, 8	1.57	4.46E‐03
	Ociad1	NM_001159889	OCIA domain containing 1	1.18	1.12E‐02
Underexpressed in null	Rpl29	NM_009082	Ribosomal protein L29	0.50	4.67E‐11
	Gm5089	NR_033325	Predicted gene 5089	0.20	5.34E‐07
	Rplp0	NM_007475	Ribosomal protein, large, P0	0.70	3.06E‐04
	Pcdhb9	NM_053134	Protocadherin beta 9	0.78	9.35E‐04
	Nsun7	NM_027602	NOL1/NOP2/Sun domain family, member 7	0.56	2.05E‐02

When evaluating transcriptomes by tissues and comparing WT versus null (collapsed across age), several of the same genes were over and underexpressed in the nulls (Table 2). For example, Rpl29 was underexpressed in all three tissue types; Rplp0 was only underexpressed in the cortex and Atp10d was only underexpressed in the hippocampus. However, some new genes were identified including a RIKEN cDNA described as a Y box protein 1 pseudogene, and the predicted gene Gm21541 that is the human homolog of CCL21, a high‐affinity functional ligand for chemokine receptor 7. In addition, Lcn2 was dramatically upregulated in null cortex. Interestingly, Lcn2 null mice display anxious and depressive‐like behaviors and cognitive impairment in a spatial learning task, suggesting that Lcn2 regulates emotional behaviors and cognitive functions (Ferreira et al. 2013). Although previous work has shown that Lcn2 is expressed at low basal levels in the hippocampus (Chia et al. 2011), LCN2 expression is up‐regulated in response to stress (Mucha et al. 2011), which has been linked to increased spinogenesis and spine maturation in the CA1–CA3 regions of the hippocampus. Finally, Cdhr1 was also overexpressed in null cortex, reiterating the role of cadherins in development and behavior.

**Table 2:** DGE by brain region

Tissue	Gene	Refseq	Annotation	Fold change	*P*adj
Cortex	Rpl29	NM_009082	Ribosomal protein L29	0.50	4.11E‐06
	Rplp0	NM_007475	Ribosomal protein, large, P0	0.69	1.80E‐03
	Lcn2	NM_008491	Lipocalin 2	16.38	1.84E‐18
	E130201H02Rik	NR_024324	RIKEN cDNA E130201H02 gene	2.51	2.85E‐05
	Cdhr1	NM_130878	Cadherin‐related family member 1	2.01	4.50E‐03
	Gm21541	NM_001270360	Predicted gene, 21541	3.93	3.16E‐02
Striatum	Rpl29	NM_009082	Ribosomal protein L29	0.46	3.11E‐02
Hippocampus	Atp10d	NR_003966	ATPase, class V, type 10D	2.56	8.65E‐03
	Rpl29	NM_009082	Ribosomal protein L29	0.58	4.24E‐02

When samples were collapsed across brain region and gene expression was assessed by age, the list of genes with statistically significant genotype‐dependent DGE expanded (Table 3). In addition, the numbers of genes that displayed differential genotype‐dependent profiles was greater at earlier ages. Specifically, there were 16 genes at 6 months, 33 genes at 1 month, and 47 genes at 4 days, suggesting that the consequences of loss of *Lphn3* are temporally dynamic, and consistent with characterization of ADHD as a developmental disorder. Some of the previously identified genes were also significant in this analysis, including Rpl29 at all time points and others at specific time points (Atp10d at 6 months, Lcn2 at 1 month, and Rplp0 at both 6 months and 4 days). An unclassified noncoding RNA gene, 2610507I01Rik, was significant at all three time points. Also of note was the differential expression of Serpina3 genes. Murine Serpina3 genes are part of a multigene cluster of 14 genes that are all homologs to the single human antichymotrypsin (SERPINA3) (Forsyth et al. 2003). Serpina3n and Serpina3m were both downregulated in nulls at 6 months and 1 month, whereas Serpina3f was upregulated at 1 month. As all the 14 Serpina3 genes are contiguous on murine chromosome 12, it is interesting that some were over‐ and others underexpressed in the *Lphn3* nulls. Variations in human SERPINA3 sequence have been implicated in Alzheimer's disease, and deficiency of this protein has been associated with liver disease. Mutations have been identified in patients with Parkinson disease and chronic obstructive pulmonary disease.

**Table 3:** DGE by age

Time	Gene	Refseq	Annotation	Fold change	*P*adj
6 months	Serpina3n	NM_009252	Serine (or cysteine) peptidase inhibitor, clade A, member 3N	0.28	1.31E‐27
	2610507I01Rik	NR_037964	RIKEN cDNA 2610507I01 gene	4.50	5.31E‐09
	Serpina3 m	NM_009253	Serine (or cysteine) peptidase inhibitor, clade A, member 3M	0.12	3.09E‐07
	Rpl29	NM_009082	Ribosomal protein L29	0.44	1.29E‐06
	Rplp0	NM_007475	Ribosomal protein, large, P0	0.65	5.01E‐04
	Ipcef1	NM_001170800	Interaction protein for cytohesin exchange factors 1	0.66	2.96E‐02
	Arhgap36	NM_001081123	Rho GTPase activating protein 36	0.41	3.35E‐02
	Sema3a	NM_009152	Sema domain, immunoglobulin domain (Ig), short basic domain, secreted, (semaphorin) 3A	0.52	3.45E‐02
	Gabrq	NM_001290435	Gamma‐aminobutyric acid (GABA) A receptor, subunit theta	0.43	3.45E‐02
	Slc6a3	NM_010020	Solute carrier family 6 (neurotransmitter transporter, dopamine), member 3	2.18	5.46E‐02
	Hspa1a	NM_010479	Heat shock protein 1A	1.84	1.15E‐05
	Hspa1b	NM_010478	Heat shock protein 1B	1.97	7.82E‐05
	Pttg1	NM_013917	Pituitary tumor‐transforming gene 1	4.20	1.44E‐03
	Prkcd	NM_011103	Protein kinase C, delta	1.62	3.24E‐03
	Htr2c	NM_008312	5‐hydroxytryptamine (serotonin) receptor 2C	1.59	1.67E‐02
	Atp10d	NR_003966	ATPase, class V, type 10D	2.44	2.46E‐02
1 month	Serpina3n	NM_009252	Serine (or cysteine) peptidase inhibitor, clade A, member 3N	0.35	1.25E‐07
	Serpina3 m	NM_009253	Serine (or cysteine) peptidase inhibitor, clade A, member 3M	0.10	1.31E‐07
	Shox2	NM_013665	Short stature homeobox 2	0.26	1.11E‐03
	Rpl29	NM_009082	Ribosomal protein L29	0.60	8.63E‐03
	Il33	NM_001164724	Interleukin 33	0.63	1.72E‐02
	2610507I01Rik	NR_037964	RIKEN cDNA 2610507I01 gene	0.50	3.13E‐02
	Lcn2	NM_008491	Lipocalin 2	81.32	1.99E‐60
	Tmem252	NM_183160	Transmembrane protein 252	5.22	1.15E‐11
	Serpina3f	NM_001168294	Serine (or cysteine) peptidase inhibitor, clade A, member 3F	11.17	6.40E‐10
	Cp	NM_001276250	Ceruloplasmin	2.26	1.31E‐07
	Cd14	NM_009841	CD14 antigen	3.78	1.31E‐07
	Ccl12	NM_011331	Chemokine (C‐C motif) ligand 12	6.72	4.32E‐07
	Il4ra	NM_001008700	Interleukin 4 receptor, alpha	2.71	4.64E‐07
	Lrg1	NM_029796	Leucine‐rich alpha‐2‐glycoprotein 1	4.87	2.73E‐06
	Runx1	NM_001111023	Runt‐related transcription factor 1	3.58	5.73E‐06
	Ch25 h	NM_009890	Cholesterol 25‐hydroxylase	5.15	8.13E‐05
	Socs3	NM_007707	Suppressor of cytokine signaling 3	3.33	1.03E‐04
	Acer2	NM_139306	Alkaline ceramidase 2	2.30	1.98E‐04
	Pisd‐ps3	NR_003518		1.60	2.51E‐04
	Stbd1	NM_175096	Starch‐binding domain 1	3.20	2.51E‐04
	C1s1	NM_144938	Complement component 1, s subcomponent 1	3.81	2.73E‐04
	8430408G22Rik	NM_145980	RIKEN cDNA 8430408G22 gene	3.94	2.73E‐04
	Steap4	NM_054098	STEAP family member 4	4.45	3.66E‐04
	Csf2rb2	NM_007781	Colony‐stimulating factor 2 receptor, beta 2, low‐affinity (granulocyte‐macrophage)	4.26	1.11E‐03
	Ackr1	NM_010045	Atypical chemokine receptor 1 (Duffy blood group)	1.70	1.11E‐03
	Selp	NM_011347	Selectin, platelet	5.27	1.59E‐03
	1500015A07Rik	NR_029432	RIKEN cDNA 1500015A07 gene	2.00	1.82E‐03
	Vwf	NM_011708	Von Willebrand factor homolog	2.07	2.98E‐03
	Osmr	NM_011019	Oncostatin M receptor	2.22	4.23E‐03
	Scgb3a1	NM_170727	Secretoglobin, family 3A, member 1	4.59	9.06E‐03
	Atp10d	NR_003966	ATPase, class V, type 10D	2.32	2.12E‐02
	Tnfsf8	NM_009403	Tumor necrosis factor (ligand) superfamily, member 8	4.26	2.69E‐02
	Il1r1	NM_001123382	Interleukin 1 receptor, type I	1.86	4.84E‐02
4 days	Rplp0	NM_007475	Ribosomal protein, large, P0	0.59	5.89E‐10
	Dcn	NM_001190451	Decorin	0.38	2.37E‐07
	Ogn	NM_008760	Osteoglycin	0.32	2.37E‐07
	Fmod	NM_021355	Fibromodulin	0.30	4.50E‐05
	Rpl29	NM_009082	Ribosomal protein L29	0.49	8.22E‐05
	Gpc2	NM_172412	Glypican 2 (cerebroglycan)	0.59	2.23E‐04
	Lox	NM_010728	Lysyl oxidase	0.36	6.15E‐04
	Sox11	NM_009234	SRY (sex determining region Y)‐box 11	0.69	1.14E‐03
	H19	NR_001592	H19, imprinted maternally expressed transcript	0.50	1.14E‐03
	Cldn11	NM_008770	Claudin 11	0.48	1.14E‐03
	Scube1	NM_001271472	Signal peptide, CUB domain, EGF‐like 1	0.63	1.14E‐03
	Thbd	NM_009378	Thrombomodulin	0.49	3.12E‐03
	Colec12	NM_130449	Collectin subfamily member 12	0.56	3.66E‐03
	Col1a2	NM_007743	Collagen, type I, alpha 2	0.56	4.14E‐03
	Gjb2	NM_008125	Gap junction protein, beta 2	0.45	1.06E‐02
	Islr	NM_012043	Immunoglobulin superfamily containing leucine‐rich repeat	0.55	2.56E‐02
	Lepr	NM_001122899	Leptin receptor	0.43	2.96E‐02
	Aebp1	NM_001291857	AE‐binding protein 1	0.49	2.96E‐02
	Aldh1a2	NM_009022	Aldehyde dehydrogenase family 1, subfamily A2	0.44	3.35E‐02
	Mpped1	NM_172610	Metallophosphoesterase domain containing 1	0.78	3.39E‐02
	Hddc3	NM_026812	HD domain containing 3	0.50	3.68E‐02
	Cdkn1c	NM_001161624	Cyclin‐dependent kinase inhibitor 1C (P57)	0.57	3.68E‐02
	Cd24a	NM_009846	CD24a antigen	0.76	4.13E‐02
	Lynx1	NM_011838	Ly6/neurotoxin 1	1.67	8.44E‐06
	Cdkl2	NM_001276315	Cyclin‐dependent kinase‐like 2 (CDC2‐related kinase)	1.45	3.96E‐04
	Rpl3	NM_013762	Ribosomal protein L3	1.50	6.15E‐04
	Aqp4	NM_009700	Aquaporin 4	1.40	1.14E‐03
	Vsnl1	NM_012038	Visinin‐like 1	1.38	1.14E‐03
	Creg2	NM_170597	Cellular repressor of E1A‐stimulated genes 2	1.50	1.66E‐03
	Slc24a2	NM_172426	Solute carrier family 24 (sodium/potassium/calcium exchanger), member 2	1.45	3.12E‐03
	Nrxn3	NM_001198587	Neurexin III	1.35	6.15E‐03
	Hapln1	NM_013500	Hyaluronan and proteoglycan link protein 1	1.53	6.24E‐03
	Bend4	NM_001164806	BEN domain containing 4	1.59	1.06E‐02
	Lsm11	NM_028185	U7 snRNP‐specific Sm‐like protein LSM11	1.31	1.06E‐02
	Fabp5	NM_010634	Fatty acid‐binding protein 5, epidermal	1.35	1.26E‐02
	2610507I01Rik	NR_037964	RIKEN cDNA 2610507I01 gene	2.17	1.30E‐02
	Sash1	NM_175155	SAM and SH3 domain containing 1	1.40	1.41E‐02
	Camk2a	NM_177407	Calcium/calmodulin‐dependent protein kinase II alpha	1.40	2.52E‐02
	Map1a	NM_032393	Microtubule‐associated protein 1 A	1.42	2.52E‐02
	Ednrb	NM_001136061	Endothelin receptor type B	1.29	3.10E‐02
	Kcnd2	NM_019697	Potassium voltage‐gated channel, Shal‐related family, member 2	1.30	3.31E‐02
	Cadm2	NM_178721	Cell adhesion molecule 2	1.29	3.66E‐02
	Cntn1	NM_001159647	Contactin 1	1.29	3.66E‐02
	Slc1a2	NM_001077514	Solute carrier family 1 (glial high‐affinity glutamate transporter), member 2	1.32	3.66E‐02
	Rasgrp1	NM_011246	RAS guanyl releasing protein 1	1.37	3.68E‐02
	Omg	NM_019409	Oligodendrocyte myelin glycoprotein	1.44	4.08E‐02
	Gad2	NM_008078	Glutamic acid decarboxylase 2	1.35	4.94E‐02

Other genes identified at 6 months included underexpression in nulls of both Sema3a, a member of the semaphorin family vital for normal neuronal pattern development involved in growth cone collapse and axon pruning and repulsion, and Gabrq, a subunit of the GABA(A) receptor. It is notable that two other previously identified ADHD candidate genes were overexpressed in nulls at 6 months: Htr2c (5‐hydroxytryptamine (serotonin) receptor 2c) and Slc6a3 (Dat1). Htr2c was overexpressed in both the null cortex and hippocampus, whereas Slc6a3 was overexpressed in the null striatum. Slc6a3 encodes a dopamine transporter which mediates active reuptake of dopamine from the synapse and is a principal regulator of dopaminergic neurotransmission. Variation in Slc6a3 is associated with idiopathic epilepsy, ADHD, alcohol and cocaine dependence, susceptibility to Parkinson disease, and protection against nicotine dependence (Vaughan and Foster 2013). In fact, DAT1 was believed to represent one of the first replicated relations of a candidate gene to a psychiatric disorder in children. Interestingly, many individuals with ADHD respond well to medications such as methylphenidate that block DAT1, leading to increased amounts and duration of dopamine in the synapse (Amara and Kuhar 1993).

Differential genotype‐dependent gene expression at 1 month included Runx1, a promoter of neuronal differentiation (Kobayashi et al. 2012) and Ch25 h (cholesterol 25‐hydroxylase) (Papassotiropoulos et al. 2005), both of which were upregulated in nulls. It is also interesting to note that eight of the upregulated genes in the null mouse were chemokines, cytokines, or their receptors: Ccl12, Il4ra, Socs3, Csf2rb2, Ackr1, Scgb3a1, Tnfsf8, and Il1r1. The cytokine Il33 was downregulated.

At 4 days, there was genotype‐dependent differential expression of 47 genes, over half of which have known roles in neuron structure, function, differentiation, and/or development. As a whole, several extracellular matrix and proteoglycan genes were downregulated in the null: Dcn, Ogn, Fmod, Gpc2, and Col1a2. There was also an overexpression of some cell adhesion molecules in nulls: Nrxn3, Cadm2, and Cntn1. Several plausibly relevant transporter genes were also upregulated. Slc1a2 is the principal transporter that clears the excitatory neurotransmitter glutamate from the extracellular space at synapses in the central nervous system. Glutamate clearance is necessary for proper synaptic activation and to prevent neuronal damage from excessive activation of glutamate receptors. Mutations in and decreased expression of this protein are associated with amyotrophic lateral sclerosis (ALS) (Lin et al. 1998). Slc24a2 was also overexpressed. Homozygous mutation of Slc24a2 in mice results in changes in hippocampal synaptic plasticity as judged by a significant loss of long‐term potentiation in CA1 pyramidal neurons and deficits in motor learning and spatial working memory (Li et al. 2006). Finally, Gad2 (glutamic acid decarboxylase 2) was upregulated in the nulls. This is relevant as it catalyzes the decarboxylation of glutamate to GABA and CO_2_; null mice exhibit spontaneous (frequently fatal) seizures, increased anxiety‐like behavior, and reduced intermale aggression (Kash et al. 1997). Heterozygotes show reduced aggression.

Gene set analysis (GSA) is a powerful strategy that can be used to infer functional and mechanistic changes from high‐throughput microarray or sequencing data. GSA focuses on sets of related genes and has established major advantages over per‐gene based differential expression analyses, including greater robustness, sensitivity, and biological relevance. In this study, a new GSA method called Generally Applicable Gene‐set Enrichment (GAGE) was utilized to test whether a gene set was statistically associated with a genotype (Luo et al. 2009). Specifically, GAGE examined the fold changes of gene expression levels in the null samples versus the WT samples. A two‐sample t‐test was used to test whether the mean fold change of a target gene set was different from that of the background set.

Overall, when evaluating all null and all WT samples, there was enrichment for 34 KEGG IDs for which *P *<* *0.05 (Table 4); however, after adjustment for multiple comparisons by the Benjamini–Hochberg FDR method, adjusted *P*‐values were no longer significant. This was because the lack of true replicates meant the variability was too large to obtain significant hits. Regardless, the enriched pathways included those that would be anticipated given *Lphn3's* association with ADHD and addiction. One of the more enriched pathways was synaptic vesicle cycle with *P *=* *0.0053. Other pathways of note included the cholinergic and GABAergic synapse, SNARE interactions in vesicular transport, and amphetamine addiction.

**Table 4:** GAGE analysis

Overall WT versus null			Prefrontal cortex, WT versus null		
Description	Mean change	*P*.val	Description	Mean change	*P*.val
Adrenergic signaling in cardiomyocytes	2.93	0.0018	Synaptic vesicle cycle^a^	3.49	0.0004
Insulin secretion	2.87	0.0023	Adrenergic signaling in cardiomyocytes^a^	2.49	0.0067
Oxytocin signaling pathway	2.70	0.0037	ErbB signaling pathway^a^	2.45	0.0077
Renal cell carcinoma	2.66	0.0044	Dopaminergic synapse	2.25	0.0127
Synaptic vesicle cycle	2.60	0.0053	Oxytocin signaling pathway^a^	2.24	0.0130
Spliceosome	2.52	0.0062	MAPK signaling pathway^a^	2.22	0.0133
Glioma	2.46	0.0076	Endocytosis	2.19	0.0144
Proximal tubule bicarbonate reclamation	2.50	0.0087	Protein processing in endoplasmic reticulum	2.18	0.0149
ErbB signaling pathway	2.37	0.0094	Tight junction	2.06	0.0203
mRNA surveillance pathway	2.36	0.0098	Thyroid hormone signaling pathway^a^	2.01	0.0227
Huntington's disease	2.28	0.0116	Amphetamine addiction^a^	1.98	0.0249
Regulation of actin cytoskeleton	2.22	0.0135	Insulin signaling pathway	1.86	0.0323
Salivary secretion	2.17	0.0159	Neurotrophin signaling pathway	1.85	0.0329
Thyroid hormone signaling pathway	2.12	0.0177	Glioma^a^	1.84	0.0340
Cardiac muscle contraction	2.11	0.0185	mRNA surveillance pathway^a^	1.82	0.0352
MAPK signaling pathway	2.08	0.0190	Salmonella infection	1.82	0.0356
Lysosome	2.07	0.0199	Proximal tubule bicarbonate reclamation^a^	1.85	0.0366
cGMP‐PKG signaling pathway	2.06	0.0202	Huntington's disease^a^	1.79	0.0375
Ubiquitin mediated proteolysis	2.00	0.0230	Circadian entrainment	1.79	0.0376
GnRH signaling pathway	2.01	0.0233	Bacterial invasion of epithelial cells^a^	1.79	0.0378
Bacterial invasion of epithelial cells	1.99	0.0245	Retrograde endocannabinoid signaling	1.76	0.0398
Circadian rhythm	1.93	0.0290	Glutamatergic synapse	1.75	0.0406
Hepatitis B	1.90	0.0290	Renal cell carcinoma^a^	1.70	0.0460
Cholinergic synapse	1.80	0.0370	Nicotine addiction	1.69	0.0480
Amphetamine addiction	1.80	0.0371	cGMP‐PKG signaling pathway^a^	1.67	0.0481
SNARE interactions in vesicular transport	1.81	0.0378	Proteoglycans in cancer	1.66	0.0487
Prostate cancer	1.78	0.0385			
Gastric acid secretion	1.78	0.0392			
Aminoacyl‐tRNA biosynthesis	1.77	0.0404			
GABAergic synapse	1.73	0.0428			
Vasopressin‐regulated water reabsorption	1.72	0.0442			
Ribosome	1.70	0.0455			
Focal adhesion	1.68	0.0467			
Estrogen signaling pathway	1.66	0.0490			

a Gene sets in common with overall analysis.

We also conducted GAGE analyses for tissue and time point. When the analysis was broken out by tissue, 26 pathways were identified in the prefrontal cortex, eight in the hippocampus, and eight in the striatum (Table 4 and Table S4 summarizing all GAGE data for which *P *<* *0.05). There was overlap between 14 of the prefrontal cortical pathways and the overall analysis (indicated by highlighting in Table 4), which included the synaptic vesicle cycling and amphetamine addiction pathways. Additional KEGG pathway IDs of note in prefrontal cortex included the dopaminergic synapse, glutamatergic synapse, neurotrophin signaling pathway, and nicotine addiction. Hippocampal pathways overlapped little with the overall pathway analysis and not at all with the other tissues. Hippocampal KEGG ID terms were difficult to reconcile with roles in ADHD, addiction, or roles in neuronal development, function, or structure. The striatal pathways overlapped with the overall pathway analysis and cortical analysis at oxytocin signaling pathway. There was further overlap between cortex and striatum with retrograde endocannabinoid signaling.

When the pathways were broken out by time, there were only three pathways at 6 months, but 30 at 1 month and 27 at 4 days (Table S4 summarizing all GAGE data for which *P *<* *0.05). Nicotine addiction was notable at 6 months. Fourteen pathways overlapped between the overall analysis and 1 month analysis. Regulation of actin cytoskeleton and axon guidance pathways was enriched at 1 month. Fifteen pathway terms overlapped at 4 days with the overall analysis including GABAergic synapse and synaptic vesicle cycle. While there was no overlap between the 6 month terms and the 1 month and 4 day terms, the 1 month and 4 day terms overlapped for six pathways: adrenergic signaling in cardiomyocytes, estrogen signaling pathway, glioma and GnRH signaling pathway, oxytocin signaling pathway, and retrograde endocannabinoid signaling.

In addition, we evaluated the transcriptomes for genotype‐based differential RNA processing. By mapping splice acceptor and donor sites and comparing them to identify the sites with the greatest differences in acceptor sites for a given donor site, most of these represent shifts in the distribution of alternative transcripts, as opposed to clear cases where an exon is added or deleted in the null mouse. However, after adjusting for multiple tests using the Benjamini–Hochberg procedure (with a maximum false‐discovery rate of 0.05), none of the sites were found to be statistically significant. This suggests that the disruption of *Lphn3* did not induce differential RNA splicing or significant alternative mRNA transcripts for any gene.

## Discussion

While the *LPHN3* gene has been shown to be associated with ADHD and addiction, there is still very little known about the function of this gene and how it contributes to these psychiatric conditions. The data presented in this study extend our previous work and further characterize this gene at the behavioral, biochemical, cellular, and transcriptional levels. Mice null for the *Lphn3* gene demonstrate exaggerated reward‐seeking behavior, but have intact working memory capacity and locomotor coordination. At the neuronal level, the data indicate that cortical neurons display enhanced neurite outgrowth, and show dramatic yet temporally restrained changes in gene expression. These data are consistent with the idea that ADHD is characterized by a delay in cortical maturation, and highlight transcriptional pathways that might mediate these effects. The findings demonstrate the need to further investigate *Lphn3* function in the developmental timing of altered gene expression and its effects on neuronal structure/function during brain development.

### Behavior

While there were differences in gene expression within the prefrontal cortex between null and WT mice, it is important to note that there were no differences in performance in the working memory task, which is dependent upon the prefrontal cortex (Funahashi and Kubota 1994; Sloan et al. 2006). This could suggest that differences in expression of these genes do not render the prefrontal cortex dysfunctional, allowing normal executive functioning to occur. However, there are several alternative explanations that may account for these inconsistent findings. First, the “information maintenance over delays” aspect of working memory is only one of several that tap into prefrontal cortical functioning. Thus, it is possible that the use of other measures of executive function, such as attention, set‐shifting, or reversal learning, would reveal deficits in the null mice. Second, all of the mice were tested in the working memory task as adults (4–6 month). The transcriptome results suggest, however, that the majority of gene expression differences occur early in life. Hence, it is possible that deficits in working memory occur prior to adulthood, but become attenuated with development. Future studies will address this issue by assessing working memory capabilities at various time points across the lifespan.

In contrast to the working memory task, there were differences in food motivation between *Lphn3* null and WT mice such that the former displayed elevated instrumental responding for food reward (appetitive motivation), particularly at higher ratios of reinforcement. While there is a wealth of literature documenting manipulations that decrease motivation to work for food or other rewards, there are very few that potentiate food motivation, indicating that the loss of *Lphn3* and the resulting changes in gene expression cause a very selective effect on motivated behavior. A recent report Gourley et al. (2010) showed that lesions of the medial orbitofrontal cortex in mice resulted in an increase in instrumental responding for food reward under a progressive ratio schedule. Hence, alterations in gene expression in the orbitofrontal cortex in null mice may have caused dysfunction in this region, which manifested as an increase in responding for food. The increased responding of the null mice on the FR schedules also parallels behavior in amphetamine‐sensitized rats, which show concurrent enhancements in striatal dopamine release (Robinson et al. 1998; Vanderschuren and Kalivas 2000; Wyvell and Berridge 2000, 2001; Mendez et al. 2009). Given that *Lphn3* null mice have elevated striatal dopamine levels (Wallis et al. 2012), the increases in food reward‐seeking behavior in these mice may be due to dopamine‐mediated enhancements in the motivational value of the food reward or willingness to overcome effort costs to obtain reward (Wyvell and Berridge 2000; Floresco et al. 2008; Salamone and Correa 2012). These results are also consistent with findings of altered motivation linked to striatal dopamine signaling in ADHD and SUD (although these conditions are usually associated with decreased rather than increased motivation for natural rewards (Paloyelis et al. 2012; Plichta and Scheres 2014; Volkow et al. 2011; Saunders and Robinson 2013; Volkow et al. 2012). There were very few gene expression differences in the striatum between null and WT mice. Given the role of the striatum in reward‐related behavior, these findings were surprising in light of the differences observed in the instrumental responding task. One possible explanation is that loss of *Lphn3* resulted in dysfunction within the prefrontal cortical‐striatal pathway, rather than in the striatum itself. Specifically, alterations in gene expression within the prefrontal cortex could have disrupted signaling within the striatum at the presynaptic level, depriving the striatum of normal input. Consistent with this idea is the fact that LPHN3 receptors, located presynaptically, are responsible for regulating neurotransmitter exocytosis. The loss of this receptor on cortico‐striatal presynaptic terminals would result in altered synaptic transmission within the striatum. Lending support to this hypothesis, neuroimaging studies report reduced functional connectivity between the prefrontal cortex and striatum in individuals diagnosed with ADHD, and treatment with methylphenidate ameliorates this reduction (Liston et al. 2011). Future studies will probe differential connectivity between brain regions in these null mice using electrophysiology and functional imaging.

In the forced swim test, *Lphn3* null mice displayed significantly greater activity levels than WT mice, with all but one null mouse failing to exhibit immobility during the entire test period. This finding is consistent with the increase in activity levels found in null mice in our previous report (Wallis et al. 2012), and provides additional evidence for their hyperactive phenotype. Finally, there were few differences in behavior between male and female mice in this study (such differences were only evident in motor coordination, for which there was no significant genotype difference). Although the present studies were not strongly powered to detect such sex differences, given that diagnoses (though perhaps not incidence) of both ADHD and SUD are more prevalent in males than females, it will be important in future studies to investigate this issue more thoroughly.

### Neuron structure and biochemistry

The in vitro analysis of neuron structure based on primary cultures of *Lphn3* null and WT neurons suggests that Lphn3 plays a significant role in neurite outgrowth. We observed increased neurite outgrowth in cortical (but not hippocampal) neurons, consistent with recent data showing that *LPHN*3 is important in synaptic development. Others have shown that loss of *LPHN3* via shRNA results in a reduction in the number of glutamatergic synapses in dissociated hippocampal cultures (O'Sullivan et al. 2012). *LPHN3* is also key in determining the connectivity rates between principal neurons in the 2‐week‐old rat cortex (O'Sullivan et al. 2014). LPHN3 interacts trans‐synaptically with FLRTs during synapse development and contributes to determining the density of synaptic terminals formed by axons of cortical pyramidal neurons.(O'Sullivan et al. 2014)**.**

The biochemical analysis of blood from null and WT mice indicates that null mice have low serum calcium levels. Typically, serum calcium levels are very tightly regulated. While one common cause of hypocalcemia is vitamin D deficiency, both null and WT mice had similar vitamin D levels. In addition, hypocalcemia leads to increased parathyroid hormone (PTH) levels (Chen and Goodman 2004); although we did not evaluate PTH in this study, PTH changes could be an indicator of the physiological relevance of the reduced calcium levels. While any direct links between serum calcium levels and intracellular calcium levels are unclear, calcium plays a major role in neurons as the trigger for neurotransmitter release and is critical for neural development and synaptic plasticity. Given this crucial role, we evaluated our transcriptome data for evidence of altered calcium signaling. Camk2a (calcium/calmodulin‐dependent protein kinase II) is overexpressed at 4 days (Table 3). We also observed decreased levels of other calcium signaling genes in the null striatum at specific time points. Nulls have threefold less Calml4 at 4 days, over twofold less Calb1, Calb2, Cacna1g, and Cacna1s at 1 month, and twofold less Camk1g and 7.5‐fold less Calcr at 6 months. With the exception of Camk2a, there were no other statistically significant genes when analyzed over all three time points and three brain regions. Future studies should focus on differences in calcium signaling or calcium levels in the brain or in neurons and evaluate striatal transcripts for changes in calcium regulating genes during development. These may be key as calbindin (Calb1 and Calb2) functions as a calcium sensor required for normal signaling of synaptically evoked calcium transients, and is involved in synaptic plasticity, long‐term potentiation, memory formation, and the regulation of exocytosis (McCann and Ames 2008). Interestingly, recent data suggest that individuals with ADHD and alcohol use disorders have low levels of serum calcium (Naude et al. 2012; Bener and Kamal 2014; Bener et al. 2014; Kamal et al. 2014).

### Transcriptome

This study provides the first comprehensive assessment of the transcriptome of an animal model lacking *Lphn3* function, and a number of genes and gene pathways were identified as altered in a brain regionally specific manner. The transcriptome analysis also indicates that the differential expression of most genes in null mice compared to WT is temporally dynamic. Early time points show the most changes in gene expression, with genotype‐dependent differences in gene expression attenuating with age. This temporal shift in altered gene expression profiles between WT and null mice was unanticipated, and likely reflects developmental delays in gene expression and subsequent brain development. It is important to note that our previous studies utilizing whole‐brain homogenates from a larger cohort of slightly younger mice (postnatal day 0) and only a few candidate genes showed changes in total brain RNA for genes including *5‐Htt, 5‐Ht2a, Dat, Drd4, Ncam, Nurr*, and *Th*. As these were not necessarily among the most statistically significant findings in this study, these potential discrepancies could be explained by multiple factors, including age, tissue source, and decreased statistical power based on smaller cohort size and many more genes.

Collectively, the findings of altered expression of cadherins and protocadherins are exciting. Activation of cadherins and protocadherins leads to changes in intracellular calcium by acting at voltage‐dependent calcium channels in the neuronal cell surface membrane and affecting signaling and intracellular calcium stores (Sheng et al. 2013). Cadherins and protocadherins play a role in the regulation of neurite outgrowth, neuronal recognition, and connectivity (Takeichi 2007). Cadherins play roles in axon guidance, and initiation of dendritic arborizations. It has been suggested that the large diversity of protocadherins indicates that they may have roles in establishing specific neuronal connections (Takeichi 2007). For example, the g‐protocadherins are expressed in subsets of neurons and recruited to synaptic regions. Mice lacking Pcdh‐g locus activity show decreased synaptic density and decreased synapse activity. Pchd‐g is required for maintenance of spinal interneurons (Wang et al. 2002). Further, cadherin‐13 (CDH13) is a cell adhesion molecule, which has been reliably associated with liability for ADHD and related neuropsychiatric conditions (Rivero et al. 2013).

We also observed overexpression of many different genes involved in neurotransmission, such as genes for GABA(A) receptor subunits and 5‐HT2c receptors as well as the gene for Gad2, which is responsible for the conversion of glutamate to GABA. Notably, with the exception of DAT1, most ADHD candidate genes previously identified in the literature were not implicated by the most significant findings from our transcriptome‐wide analysis. Many traditional ADHD candidate genes such as DDC, dopamine receptors, Th, serotonin receptors and transporters, and Snap25 were not among the most statistically significant results. In addition, assessment of the LPHN3 ligand FLRT3 revealed no changes in gene expression based on phenotype, tissue, or age. This highlights the importance of taking a transcriptome‐wide view to understand the impact of loss of *Lphn3*.

Two other transcriptome studies have been conducted in animal models of ADHD. One study assessed the Wig rat and performed microarray analysis to identify differentially expressed genes (DEGs). While they used a time point similar to that used here (4 weeks), they pooled brain regions (Hirano et al. 2008). This dataset overlapped with ours at genes involving other neuropsychiatric disorders and synaptic vesicular transport. Another study evaluated prefrontal cortical and striatal transcriptional responses to methylphenidate (MPH) in spontaneously hypertensive rats (an established animal model of ADHD) by microarray (dela Pena et al. 2014). In comparison to our study, this group found that treatment with MPH resulted in differential expression of 30 genes in the prefrontal cortex and 306 genes in the striatum, of which 252 were downregulated. Of those that were downregulated, many were cell adhesion molecules including Pcdh10. *Lphn3* null mice in this study also show changes in cell adhesion molecules including other protocadherins and cadherins. For example, in Pcdhgb8 was overexpressed in the *Lphn3* nulls, Cdhr1 was overexpressed specifically in the cortex, and Pcdhb9 was underexpressed. In our study, overexpression of some additional cell adhesion molecules (Nrxn3, Cadm2, and Cntn1) was observed in nulls at 4 days. In general, both the MPH study and the current *Lphn3* study suggest altered synaptic and neuronal plasticity.

While the results of the GAGE analysis were not statistically significant, they highlight pathways such as synaptic vesicle cycle, cholinergic and GABAergic synapses, SNARE interactions in vesicular transport, and amphetamine addiction, that differ between *Lphn3* null and WT mice. Similar pathway analyses have been done after GWAS studies of ADHD and drug dependence. The results of these GWAS studies further indicate the importance of neurite outgrowth and calcium signaling. For example, analysis of the 85 most significant SNPs associated with ADHD based on GWAS studies was performed. While LPHN3 was not among these genes (as it was identified through linkage and candidate gene studies), 44 of the 85 genes fell under “Neurological disease” based on Ingenuity pathway analysis (Poelmans et al. 2011). Analysis of the top GWAS genes by BiNGO bioinformatics revealed that gene ontology processes, (calcium) ion binding, and hexokinase activity were significantly enriched. Further, 45 of the 85 genes were involved in directed neurite outgrowth, and 21 of these were also involved in (calcium) ion binding. In a separate study, gene set enrichment analysis was applied to existing genetic association results to identify pertinent pathways to alcohol dependence; the calcium signaling pathway showed the most significant enrichment of associations (21 genes) (Li et al. 2014). GWAS of opioid dependence is also associated with genes in calcium and potassium signaling pathways (Wang et al. 2014). Another group performed GSA using data from the Study of Addiction: Genetics and Environment (SAGE) and results demonstrated a potential role of the ‘synthesis and degradation of ketone bodies’ and ‘neuroactive ligand–receptor interaction’ pathways (Biernacka et al. 2013).

### Potential interpretations and concluding remarks

The fact that gene expression changes ameliorate over time may reflect the temporally dynamic expression of *Lphn3* itself (Arcos‐Burgos et al. 2010) and/or developmental delays in gene expression and subsequent brain development. This is in line with the concept that ADHD is associated with delayed cortical maturation, which is an area of intense study. Interestingly, the findings of delayed structural brain maturation seem, thus far, to be specific to ADHD and may be an important neuroanatomic trait (Rubia 2007). Evidence supporting this concept is building: (1) The definition of ADHD involves “age‐inappropriate” behaviors in terms of activity and attention; (2) Children with ADHD show deficits in late‐developing higher cognitive functions of inhibitory self‐control, attention, and temporal foresight; (3) ADHD symptoms frequently ameliorate with age; (4) Structural imaging studies show decreased size of cortico‐striatal regions; (5) Functional imaging shows decreased brain activation in the same areas (Rubia et al. 2000); (6) Longitudinal neuroimaging data suggest that ADHD in childhood is characterized by a delay in cortical maturation and that different clinical outcomes are associated with different developmental trajectories in adolescence and beyond (Shaw et al. 2007, 2008; Shaw and Rabin 2009). Further, maturational delays in the cortex may be the result of modified neurite outgrowth as observed in *Lphn3* null neurons. The trajectory of cortical maturation during childhood is quite complex, but in general, it first grows thicker, then thins and matures over time (Shaw et al. 2008). While speculative, the current data are consistent with the idea that altered *LPHN3* function during development would produce enhanced neurite outgrowth at early ages, resulting in delayed thinning and maturation.

**Figure 3:**
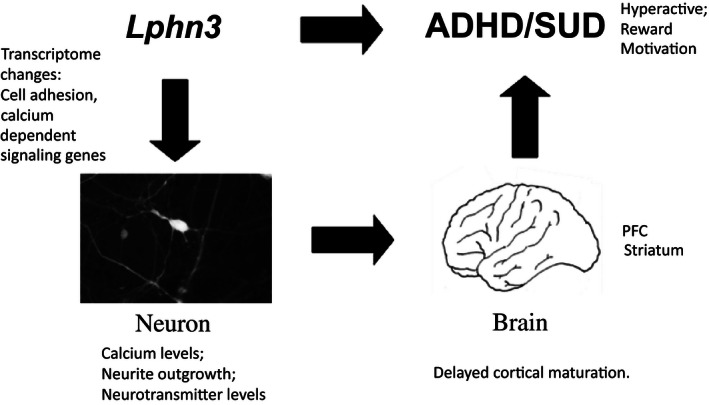
Model of *Lphn3* function. When considering genetic susceptibilities for a seemingly complex trait, such as a behavioral disorder, the rationale is fairly straightforward. We expect that genetic variation will lead to variation at the cellular level, which will subsequently lead to variation at the level of a system either due to changes during system development or in the adult organism. Such changes will result in altered behaviors. For ADHD (and addiction), a number of candidate genes have been studied, including *LPHN3*. Collectively, our data and reports in the literature indicate that loss of *Lphn3* leads to developmentally dynamic alterations in the transcriptome and suggest that cell adhesion molecules (including cadherins and protocadherins) and calcium‐dependent signaling proteins (such as calcitonin receptor, Camk1 and Camk2, voltage‐dependent calcium channels, and calbindins) are affected. At the cellular level, this affects calcium which in turn affects neurite outgrowth and neurotransmitter levels. These neuronal changes in structure and function are anticipated to affect the brain as a whole. Indeed, gross differences have been observed in the human brain between individuals with and without ADHD, including several lines of evidence for delayed cortical maturation. Finally, the alterations in neuron and brain structure/function are expected to result in behavioral changes; and in fact, we see changes in behavior in *Lphn3*‐mutant mice that encompass both activity levels and reward motivation.

As described in the Introduction, variation in *LPHN3* is associated with both ADHD and SUDs in human populations. However, this genetic variation in humans does not include null mutations in *LPHN3* (Domene et al. 2011) as in the mouse model, and hence the *Lphn3* null mouse does not have robust construct validity as a model for ADHD or SUD. That said, *Lphn3* null mice do display some phenotypes reminiscent of these disorders, and hence we propose a working model to help foster new hypotheses for testing such that we may better understand the function of *LPHN3* in development and disease (Fig. 3). According to this model, loss of *Lphn3* leads to developmentally dynamic alterations in the transcriptome, particularly in cell adhesion molecules and calcium‐dependent signaling proteins. At the cellular level, this affects calcium which in turn affects neurite outgrowth and neurotransmitter levels. This concept is supported in the literature as changes in intracellular calcium levels occur in neurons in response to activation of cell adhesion molecules such as cadherins (Sheng et al. 2013). Calcium signaling is a major determinant of homeostatic synaptic plasticity and strength (Zhao et al. 2011), and plays a critical role in all aspects of neuronal development including neurite elongation. These neuronal changes in structure and function result in the gross differences that have been observed in the human brain between individuals with and without ADHD, including several lines of evidence for delayed cortical maturation (Rubia et al. 2000; Rubia 2007; Shaw et al. 2007, 2008; Shaw and Rabin 2009). Finally, the alterations in neuron and brain structure/function lead to changes in behavioral activity levels and reward motivation. Hence, we have provided a strong foundation for future research concerning the timing of developmental gene expression and the maturational delay of the cortex. Our work should further motivate assessment of the role of *Lphn3* and cell adhesion molecules in the developing brain, and their effects on the role of calcium signaling in development and behavior.

## Conflict of Interest

None declared.

## Supporting information


**Table S1**. Mouse cohorts; genotype, gender, and age.
**Table S2**. Blood chemistry, complete blood counts, and manual differential.
**Table S3**. Lphn3 transcriptome experimental factors.

**Table S4**. GAGE analysis data *P* < 0.5.
