# Supplementary material for: Behavioral and transcriptomic profiling of mice null for Lphn3, a gene implicated in ADHD and addiction
Source: Mol Genet Genomic Med. 2016 Mar 4;4(3):322–43. doi: 10.1002/mgg3.207 (PMC4867566; doi:10.1002/mgg3.207)
Supplement: Supplementary file 1 — Table S1. Mouse cohorts; genotype, gender, and age. Table S2. Blood chemistry, complete blood counts, and manual differential. Table S3. Lphn3 transcriptome experimental factors. [file MGG3-4-322-s001.pdf]

SUPPLMENTARY INFORMATION:

Supplementary Table S1. Mouse Cohorts; Genotype, Gender and Age

Assay	Null		WT	
	Gender	Age	Gender	Age
Instrumental responding for food	M	137 days	M	137 days
	M	98 days	M	98 days
	M	82 days	M	82 days
	M	118 days	M	118 days
	M	123 days	M	118 days
	M	175 days	M	161 days
			M	213 days
			M	82 days
	F	144 days	F	137 days
	F	212 days	F	212 days
	F	213 days	F	213 days
	F	82 days	F	82 days
	F	98 days	F	82 days
	F	128 days	F	169 days
	F	147 days	F	175 days
	F	115 days	F	116 days
	F	120 days	F	98 days
	F	118 days	F	82 days
Working memory task	M	82 days	M	82 days
	M	98 days	M	82 days
			M	82 days
	F	82 days	F	82 days
	F	98 days	F	82 days
			F	98 days
			F	82 days
Rotarod	M	129 days	M	129 days
	M	129 days	M	178 days
	M	178 days	M	178 days
	M	178 days		

	F	93 days		F	87 days
	F	93 days		F	97 days
	F	96 days		F	97 days
	F	129 days		F	129 days
	F	178 days		F	178 days
Forced Swim	M	129 days		M	129 days
	M	129 days		M	178 days
	M	178 days		M	178 days
	M	178 days			
	F	93 days		F	87 days
	F	93 days		F	97 days
	F	96 days		F	97 days
	F	129 days		F	129 days
	F	178 days		F	178 days
	F	194 days		F	225 days
	F	225 days		F	225 days
CBC and Chemistry	F	4 mo		F	4 mo
	F	4 mo		F	4 mo
	F	4 mo		F	4 mo
	F	5 mo		F	4 mo
	F	5 mo		F	5 mo
				F	5 mo
Ca and Vitamin D	M	4 mo		M	4 mo
	M	4 mo		M	4 mo
	M	6 mo		M	6 mo
				M	6 mo
	F	6 mo		F	6 mo
	F	6 mo		F	8 mo
	F	6 mo		F	8 mo
	F	6 mo			
	F	6 mo			
	F	8 mo			
	F	8 mo			

Supplementary Table S2. Blood Chemistry, Complete Blood Counts and Manual Differential.

Shading in the null column indicates values that fell outside of the 90% confidence intervals established on the basis of a cohort 47 WT mice. Shading in the WT column indicates values that differed significantly from null values.

	Units	90% CI (N=47)	AVG Null (N=5)	AVG WT (N=6)
**Chemistry**				
GLU	mg/dL	110-260	170.6	162.60
BUN	mg/dL	15-34	23	22.00
CREA	mg/dL	0.1-0.3	0.2	0.20
NA	mmol/L	148-156	148.8	151.50
K	mmol/L	6.3-9.9	8.2	8.22
CL	mmol/L	105-114	108	107.00
CA	mg/dL	9.50-10.63	9.38	9.96
ALB	g/dL	2.4-3.4	3.22	3.20
T Protein	g/dL	4.8-5.8	5.36	5.27
T Bili	mg/dL	0.10-0.31	0.366	0.19
ALT	U/L	16-131	49.8	51.83
AST	U/L	36-149	150.6	75.60
GGT	U/L	0-3	0	0.17
PHOS	mg/dL	7.6-12.0	10.3	8.78
**Complete Blood Count**				
WBC	K/µL	4.85-12.35	11.122	8.07
RBC	M/µL	8.80-10.92	9.534	10.04
HGB	g/dL	13.5-16.3	14.52	15.07
HCT	%	43.3-52.3	46.68	48.22
MCV	fL	45.5-52.2	49.02	48.03
MCH	pg	14.2-15.7	15.24	15.02
MCHC	g/dL	28.0-32.1	31.1	31.23
RDW	%	17.6-22.7	20.36	20.98
PLT	K/µL	916-1671	1195.6	1314.50
MPV	fL	6.1-6.7	6.66	6.67
**Manual Differential**				

NEUT ABS	K/µL	0.27-3.18	1.16	1.17
LYMPHS ABS	K/µL	3.56-10.67	9.388	6.35
MONOS ABS	K/µL	0.07-1.10	0.474	0.48
EOS ABS	K/µL	0.00-0.45	0.1	0.08
BASOS ABS	K/µL	0.00-0.00	0	0.00
SEGS	%	2-39	10.6	12.67
BANDS	%	0-3	0.4	1.17
LYMPHS	%	56-95	83.8	79.17
MONOS	%	1-13	4.4	6.00
EOS	%	0-5	0.8	1.00
BASOS	%	0-0	0	0.00
ATYP LYMPHOS	%	0-0	0	0.00

Supplementary Table S3. Lphn3 Transcriptome Experimental Factors

Factor	Factor Description	Levels
Genotype	Lphn3 gene	WT<br>Null
Time	Age, time since birth	4 days<br>1 month<br>6 months
Tissue	Brain region	PFC<br>striatum<br>hippocampus
